# Ozone Therapy Attenuates NF-κB-Mediated Local Inflammatory Response and Activation of Th17 Cells in Treatment for Psoriasis

**DOI:** 10.7150/ijbs.41940

**Published:** 2020-04-06

**Authors:** Jinrong Zeng, Li Lei, Qinghai Zeng, Yuying Yao, Yuqing Wu, Qinxuan Li, Lihua Gao, Hongjiao Du, Yajie Xie, Jinhua Huang, Wenbin Tan, Jianyun Lu

**Affiliations:** 1 Department of Dermatology, Third Xiangya Hospital, Central South University, Changsha, Hunan, China;; 2 XiangYa School of Medicine, Central South University, Changsha, Hunan, China;; 3 Department of Cell Biology and Anatomy, School of Medicine, and; 4 Department of Biomedical Engineering, College of Engineering and Computing, University of South Carolina, Columbia, South Carolina

**Keywords:** ozone therapy, NF-κB, TLR2, Th17, psoriasis

## Abstract

Ozone therapy has been widely used to treat many skin diseases, including infections, allergic dermatosis, and skin ulcers. However, its efficacy as a treatment for psoriasis is unclear. In this study, we explored the clinical efficacy and the underlying molecular mechanisms of ozone therapy on psoriasis. We found that topical ozone treatment significantly decreased patients' psoriasis area and severity index (PASI) scores and the expression of psoriasis-associated cytokines in their peripheral blood CD4^+^ T cells. In the IMQ-induced psoriasis mouse model, topical ozone treatment significantly inhibited the formation of IMQ-induced psoriasis-like lesions and the expression of psoriasis-associated inflammatory factors. High-throughput sequencing confirmed that IMQ-induced activation of toll-like receptor 2 (TLR2)/ nuclear factor-κB (NF-κB) signaling pathway was significantly suppressed in psoriasis-like lesions after topical ozone treatment. Furthermore, the activation of spleen T helper (Th) 17 cells was blocked in the mouse model; this was associated with the downregulation of cytokines and NF-κB pathways upon topical ozone treatment. Ozone therapy can attenuate local inflammatory reactions and the activation of Th17 cells in psoriasis by inhibiting the NF-κB pathway. Our results show that ozone therapy is effective in treating psoriasis. We recommend further evaluations for its clinical applications.

## Introduction

Psoriasis vulgaris is a long-lasting immune-mediated inflammatory cutaneous disease that is characterized by red, itchy, and scaly skin patches. Patients generally suffer disfiguration, disability, and associated comorbidities 1. Environmental risk factors, such as microbial infections, obesity, and exposure to ultraviolet radiation, can trigger the onset of the disease in patients with latent psoriatic genetic susceptibility 2. Plasmacytoid dendritic cells (pDCs) have been identified as inducers in the inflammatory cascade in psoriatic plaques 3. Local pDCs in psoriatic skin lesions can activate and induce the differentiation of T helper (Th) cells into Th17, Th1, and Th22 subsets by producing IL-23, IL-12, IL-6, and tumor necrosis factor (TNF)-α 4, 5. Evidence has shown that proportions of Th1 and Th17 cells in the skin lesions and peripheral blood of psoriatic patients are significantly increased as compared with normal subjects; Th2 cells and their associated cytokines; including IL-4, IL-10, and IL-13, show decreased proportions 6, 7. Therefore, the blocking of pathogenic T cell activation, particularly the Th17 subset, has led to many remedies, such as assecukinumab 8, ixekizumab 9, and brodalumab 10. However, problems related to biological agents, such as the single effect, high costs, and drug resistance, are major concerns to many patients and physicians. In addition, multiple inflammatory cytokine-stimulated NF-κB pathways are constitutively activated in psoriatic epidermis, resulting in hyperproliferation of keratinocytes 11, 12. Activation of toll-like receptor 2 (TLR2) in keratinocytes can lead to the nuclear translocation of NF-κB and release of the proinflammatory cytokines TNF-α and IL-8 13. Microorganisms and their components and pathogen-associated molecular patterns (PAMPs) can trigger TLR2 to induce immune system activation 14. Therefore, targeting the TLR2/NF-κB pathway is a potential novel therapeutic strategy.

Ozone was first applied clinically as a sterilizing agent due to its strong oxidizing property. It has been widely used to treat more than 50 different pathological conditions, including infectious skin diseases 15-18, allergic diseases 19, 20, erythema scaly diseases 21, 22, wound healing, and ulcer recovery 23. The mechanisms of ozone's action may underlie antimicrobial effects, immunoregulation, antioxidant defenses, epigenetic modification, biosynthesis, analgesics, and vasodilation 24. Current ozone medical preparations for dermatology fall into the following primary classifications: ozone hydrotherapy, topical ozonated oil, ozone autohemotherapy (OAHT), and ozone gas cavity/acupoint injection 24. Recent studies have shown that a precise control of ozone concentrations can induce the production of various cytokines, such as IFN-γ, IL-6, and TNF-α 25. Ozone can induce and activate the body's antioxidant enzyme system to produce free radical scavenging agents, remove some of the free radicals generated by inflammatory reactions, and interfere with the production of inflammatory factors during disease development 26. However, the exact mechanisms of ozone therapy in treating diseases need to be further elucidated.

In this study, we evaluated the therapeutic efficacy of a short-term ozone treatment for psoriatic patients. We investigated potential mechanisms of topical ozone therapy for psoriasis using the imiquimod- (IMQ) induced psoriasis-like mouse model. We found that ozone therapy attenuated inflammatory responses in psoriasis by inhibiting the NF-κB pathway. Our results show that ozone therapy is a safe and effective treatment for psoriasis and is worthy of further clinical evaluations and applications.

## Materials and Methods

### Patients

This study was approved by the institutional review board (IRB) of the Third Xiangya Hospital, Central South University, Changsha, Hunan, China. A total of 10 psoriatic patients diagnosed with psoriasis vulgaris were enrolled in the study, and written consent forms were signed by all subjects. Clinical information on the patients is shown in Supplementary Table 1. PASI scores were used to assess disease activity. Study inclusion criteria were for patients between the ages of 18 and 60 years old and with psoriasis vulgaris diagnosed by pathologic examinations. Exclusion criteria included being allergic to ozonated water or oil; pregnancy or breastfeeding; severe systemic diseases; and having received corticosteroids, vitamin D3 derivatives, immune inhibitors, biological therapy, or oral retinoids within the previous 2 weeks.

### Mice

The BALB/c mice were purchased from Hunan SJA Laboratory Animal Co., Ltd. At the age of 6 weeks, female mice were all adaptively fed for 1 week and used for all experiments. All animals were raised and handled in the animal experiment center of Central South University in strict accordance with relevant laws and institutional guidelines. All animal procedures were approved and supervised by the Medicine Animal Care and Use Committee of the Third Xiangya Hospital of Central South University.

### Topical Ozone Therapy

All participants were treated with an ozonated water shower (3.0±1.5 mg/L, HZ-2601B, Hunan Health Care Technology, Changsha, China) for 15 minutes, once per day, then treated with topical ozonated oil (20160522, with an approximate peroxide value of 2,000-2,400 mmol-equivalent/kg, Hunan Health Care Technology, Changsha, China) twice per day, for 14 days.

### Evaluation of Clinical Photographs and Reflectance Confocal Microscope Images of Skin Lesions

All subjects received free ozone therapy only; they did not receive any other treatments and drugs during the trial. The intervention lasted 14 days. Clinical photographs, PASI scores, and RCM images were assessed by the same professional physicians in order to score disease severity before and after treatments. PASI scores included the area of skin lesions, erythema, scaling, and thickening, according to the literature 27. Each subject was assessed by RCM images from three different skin lesion sites. The total RCM scanned thickness of the skin was 51 layers × 3.05 µm (vertically) in each layer. Under RCM, epidermal thickness and infiltrated inflammatory cells were also evaluated prior to and post-treatment.

### IMQ-Induced Mouse Model of Psoriasis and Ozone Intervention

Female BALB/c mice (aged 6-8 weeks) were fed under suitable conditions. The mice were smeared daily with a topical 5% IMQ cream (Sichuan Med-Shine Pharmaceutical Co., Ltd., H20030128, Sichuan, China) on their shaved dorsal skins for 7 consecutive days. Mice in the control group were treated with the same quantity of the vehicle cream. All IMQ mice were randomly divided into three groups: the nonintervention group (IMQ group), the ozone-treatment group (IMQ+Ozone), and the vehicle cream-treatment group (IMQ+Vehicle). The ozone-treatment group was treated with ozonated water (HZ-2601B, Hunan Health Care Technology Co., Ltd., Changsha, China) for 15 minutes once per day, then treated with topical ozonated oil (20160522, Hunan Health Care Technology Co., Ltd., Changsha, China). The vehicle cream-treatment group received tap water and base oil at the same frequency. The intervention lasted for 7 days. Clinical photographs and PASI scores were collected in order to evaluate the phenotypic characteristics. At the 7th day, all mice were sacrificed to collect skin lesions, spleen tissues, and lymph nodes.

### Isolation of CD4^+^ T Cells

Peripheral blood mononuclear cells (PBMCs) were separated from peripheral blood of patients before and after treatment by centrifugation using a density gradient medium (GE Healthcare, Chicago, IL, USA). CD4^+^ T cells were isolated by a positive selection using Miltenyi beads according to the manufacturer's instructions (Miltenyi Biotec, Bergisch Gladbach, Germany). Next, the isolated CD4^+^ T cells were collected for subsequent experiments. In the mouse experiment, CD4^+^ T cells were purified from pooled single-cell suspensions of spleen using a mouse CD4^+^ T cell isolation kit from Miltenyi Biotec (Bergisch Gladbach, Germany).

### Flow Cytometry

Surface markers, cytokines, and transcriptional factors were detected using an FACSCanto II cell analyzer (BD Biosciences, San Jose, CA, USA). For cytokine detection, isolated cells were stimulated *in vitro* for 4 h with phorbol 12-myristate 13-acetate (PMA) and ionomycin (Sigma-Aldrich, St. Louis, MO, USA) with the addition of GolgiPlug (BD Biosciences, San Jose, CA, USA) to promote the release of cytokines. Subsequently, the treated cells were incubated with antibodies against surface markers on ice for 30 min in the dark. For intracellular staining, cells were fixed and permeabilized with an eBioscience forkhead box P3 (FOXP3) transcription factor staining buffer set (catalog No. 00-5523, San Diego, CA, USA) and then stained with fluorescent antibodies for an additional 30 min on ice in the dark. Items were collected and analyzed using the FlowJo software (FlowJo LLC, Ashland, OR, USA). The following antibodies were obtained from BioLegend (San Diego, CA, USA) and used in this study: FITC anti-mouse IFN-γ (catalog No. 505805), Alexa Fluor 647 anti-mouse IL-17A (catalog No. 506911), PE anti-mouse IL-4 (catalog No. 504103), PE anti-mouse FOXP3 (catalog No. 126403), PerCP/Cy5.5 anti-mouse CD4 (catalog No. 100540), and FITC anti-mouse CD3 (catalog No. 5100203). Phycoerythrin (PE) anti-mouse IL-4 was obtained from BD Biosciences (catalog No. 504103, San Jose, CA, USA) and APC anti-mouse CD25 was obtained from eBioscience (catalog No. 102011, San Diego, CA, USA).

### qPCR

Total RNA was extracted from cells or skin tissues using TRIzol according to the manufacturer's instructions (Thermo Fisher Scientific, Waltham, MA, USA). The mRNA was reverse-transcribed with the PrimeScript^®^ RT reagent kit (Takara Biomedical Technology Co., Ltd., Kusatsu, Shiga, Japan) with 1 μg of total RNA in each reaction. The reaction mixture for real-time PCR contained 2 μL of cDNA, 10 μL of SYBR Premix Ex Taq™ (Takara Biomedical Technology Co., Ltd., Kusatsu, Shiga, Japan), and 400 nM of sense and antisense primers for a final volume of 20 μL. The qPCR was performed on a LightCycler^®^ 96 (Roche, Rotkreuz, Switzerland) thermocycler. The quantity of gene expression was calculated using the 2^-ΔCt^ methods and normalized to glyceraldehyde-3-phosphate dehydrogenase (GAPDH). Primers are shown in Supplementary Table 2.

### Western Blotting

CD4^+^ T cells were lysated and proteins were extracted using a nuclear extraction reagent (Boster Biological Technology, Pleasanton, CA, USA). Proteins were quantified by the Bradford reagent (Thermo Fisher Scientific, Waltham, MA, USA), followed by 12% vertical dodecyl sulfate-polyacrylamide gel electrophoresis. Proteins were then transferred into a polyvinylidene difluoride (PVDF) membrane (Sigma-Aldrich, St. Louis, MO, USA). The PVDF membrane was blocked in 5% skim milk for 1 h at room temperature, then incubated with an antibody against P65 (GB11142, 1:1000, Wuhan Servicebio Technology Co., Ltd., Wuhan, China) or P50 (ab7971, 1:5000, Abcam, Cambridge, MA, USA) for 12-16 h at 4℃ , and followed by incubating with a mouse anti-rabbit IgG antibody (H&L) (GenScript, Piscataway, NJ, USA). Proteins were detected with an enhanced chemiluminescence (ECL) western blot detection kit (Thermo Fisher Scientific, Waltham, MA, USA). Quantification of P65 and P50 was normalized to GAPDH by densitometry.

### Histological Analysis

Skin tissues from all patients and mice were fixed in formalin and embedded in paraffin (Wuhan Servicebio Technology Co., Ltd., Wuhan, China). Sections (6 µm) were stained with hematoxylin and eosin and stored at room temperature. Epidermal thickness and infiltrating inflammatory cells were assessed.

### Immunohistochemical Staining

Sections (6 µm) were stained with P50 (catalog No. BS1249, Bioworld Technology Co., Ltd., Nanjing, China), P65 (catalog No. 10745-1-AP, Proteintech, Rosemont, IL, USA) and TLR2 antibodies (catalog No. ab213676, Abcam, Cambridge, MA, USA) according to the manufacturers' instructions. Image analysis was performed using a fluorescent microscope and Leica Qwin Std analysis software (Leica, Wetzlar, Germany).

### High-Throughput Sequencing

Transcriptome profiles of the left and right sides of the skin lesions from self-control mouse models and lesions from the mouse dorsal skins in the control group and the IMQ group were obtained. Briefly, total RNA was extracted from these skin samples; the mRNA was enriched, fragmented and used for the cDNA synthesis. The cDNA fragments were amplified by PCR, and the size and quality of sequencing library were determined using an Agilent 2100 Bioanalyzer (Agilent, Santa Clara, CA, USA). The library was sequenced using a HiSeq X Ten high-throughput sequencing platform (Illumina Inc., San Diego, CA, USA). The differentially expressed genes among the selected samples were analyzed by Kyoto Encyclopedia of Genes and Genomes (KEGG) pathway enrichment analysis.

### Statistical Analysis

All of the diagrams and graphs reporting cumulative data were generated using a GraphPad Prism 6.0 (GraphPad Software, San Diego, CA, USA). The data are represented as means ± standard error of the mean (SEM). Distributions of the means were analyzed with nonparametric tests (SPSS 18.0, IBM, Armonk, NY, USA). Differences in individual treatments were analyzed by paired *t* tests. Statistical significance (*P < 0.05, **P < 0.01, ***P < 0.001) was assessed using a 2-tailed unpaired Student *t* test for comparisons between 2 groups and 1-way analysis of variance (ANOVA) with relevant post hoc tests for multiple comparisons.

## Results

### Topical ozone treatment improves the condition of skin lesions in psoriasis

**Figure 1:**
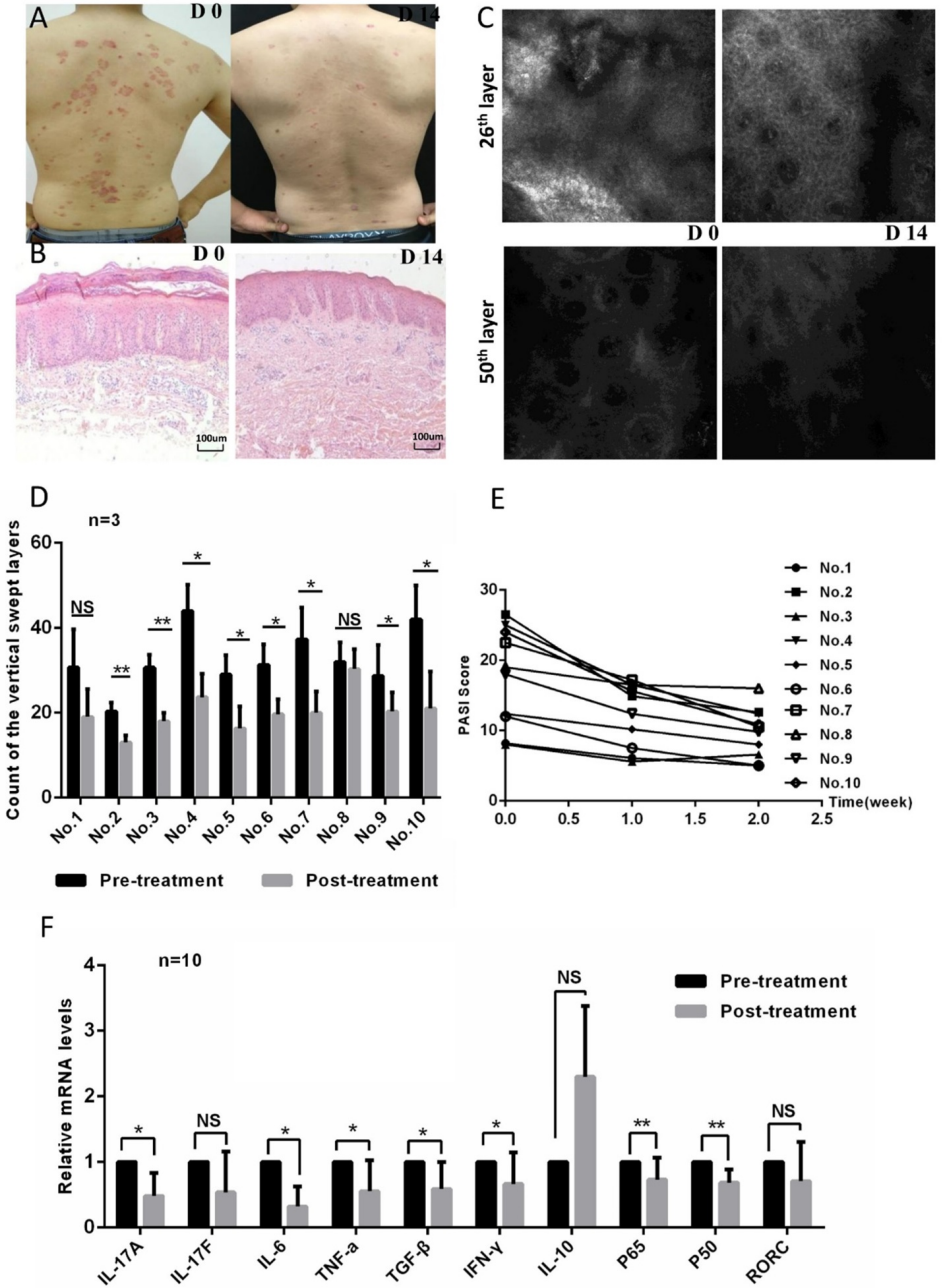
** Topical ozone treatment improves the pathological conditions of psoriatic skin lesions. (a)** Clinical photographs of a psoriatic skin lesion on days 0 (D0) and 14 (D14) with an ozone therapy. **(b)** HE staining of psoriatic skin lesion before and after treatment. **(c)** Evaluation of RCM images showing the 25th and 50th scanning layers before and after treatment. **(d)** Statistical analysis of vertical swept layers of quantitative RCM images for an assessment of thickness of epidermis; **(e)** PASI scores for all participants. **(f)** Quantitative PCR to detect expression levels of cytokines and transcriptional factors in CD4^+^ T cells from peripheral blood of psoriasis patients before and after treatment. Note: * = *P* < 0.05; ** = *P* < 0.01; *** = *P* < 0.001; NS = no statistical significance.

In order to evaluate the efficacy of ozone therapy on psoriasis, we enrolled ten patients with a diagnosis of psoriasis via cutaneous histopathology in this study. Each patient's psoriatic condition was determined using a psoriasis area and severity index (PASI) score, which was assessed four times over two weeks. In addition, clinical photographs, reflectance confocal microscopy (RCM), and hematoxylin and eosin (HE) staining were used to evaluate each patient's pathological characteristics before and after treatment. The patients' psoriatic skin lesions improved significantly after ozone therapy; clinical and histological improvements were evident in patients (Figure 1a and b). There were obvious attenuations of inflammatory erythema and scales showing in clinical photographs (Figure 1a). Histology and RCM images showed that the epidermis was significantly thinner and that infiltrating inflammatory cells had decreased after 14 days ozone treatment as compared to before treatment (Figure 1b-d). Correspondingly, the PASI scores dropped significantly after the 14-day treatment as compared with baselines (Figure 1e). In order to investigate the potential mechanisms of ozone action on psoriasis, we assessed the expression levels of common psoriatic-associated cytokines and transcription factors in CD4^+^ T cells from the patients' peripheral blood using quantitative real-time PCR (qPCR). Being expected, the expression levels of IL-17a, IL-6, TNF-α, transforming growth factor (TGF)-β, IFN-γ, and NF-κB were down-regulated after ozone-treatment as compared with prior to treatment (Figure 1f). The expression levels of IL-17f and the Th17-cell-specific transcription factor retinoid-related orphan nuclear receptor c (RORc) decreased after ozone treatment, but the decrease had no statistical significance (Figure 1f). The expression of IL-10 increased after ozone treatment, but not significantly. These results were consistent with the clinical efficacy. Taken together, our results demonstrate that topical ozone treatment can improve the condition of psoriatic skin lesions in patients by an inhibition of inflammatory processes.

### Inhibition of IMQ-induced psoriasis-like phenotypes by topical ozone treatment

**Figure 2:**
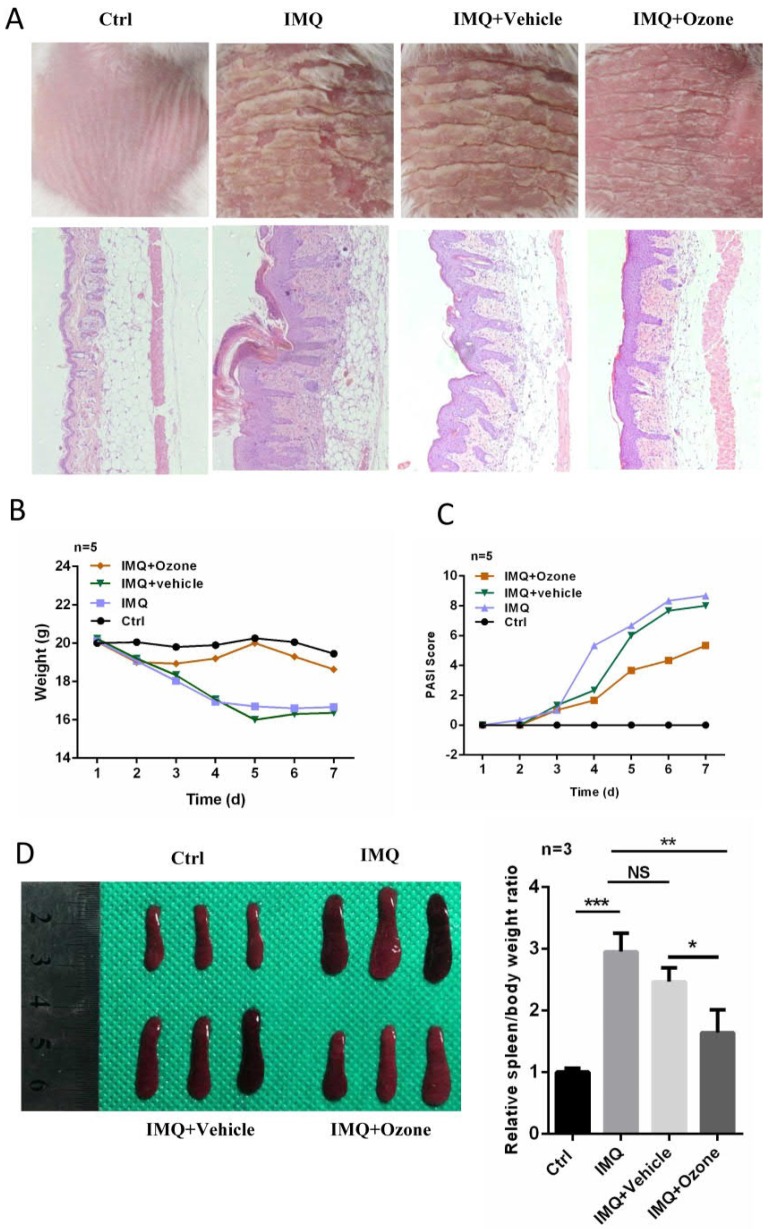
** Topical ozone treatment significantly inhibits IMQ-induced psoriasis-like lesions in mouse skin.** Mice were randomly divided into four groups: Ctrl, IMQ, IMQ+Vehicle, and IMQ+Ozone. **(a)** The skin lesions and histological features among different treatment groups. **(b)** Changes of body weight and **(c)** PASI scores for mice in different treatment groups. **(d)** Spleen and spleen-to-body-weight ratio in different treatment groups. Note: * = *P* < 0.05; ** = *P* < 0.01; *** = *P* < 0.001; NS = no statistical significance.

In order to further evaluate the therapeutic efficacy of topical ozone on psoriasis, we used IMQ to induce psoriasis-like lesions on dorsal skins of BALB/c mice 28. Daily application of topical ozone resulted in a significant inhibition of IMQ-induced psoriasis-like lesions as compared to the vehicle-treatment group (water + base oil) (Figure 2a). The topical ozone treatment prevented IMQ-induced weight loss (Figure 2b) and improved PASI scores (Figure 2c) in the IMQ-induced psoriatic mice. Previous studies 6 have shown that IMQ can cause enlargement of the spleen in this mouse model. We found that topical ozone treatment resulted in a significant inhibition of the increased spleen-to-body-weight ratio as compared with the no-treatment or vehicle-treatment groups (Figure 2d). These results demonstrate that topical ozone treatment can inhibit IMQ-induced psoriasis-like phenotypes in this mouse model.

### Inhibition of IMQ-induced psoriasis-like inflammation by topical ozone treatment

**Figure 3:**
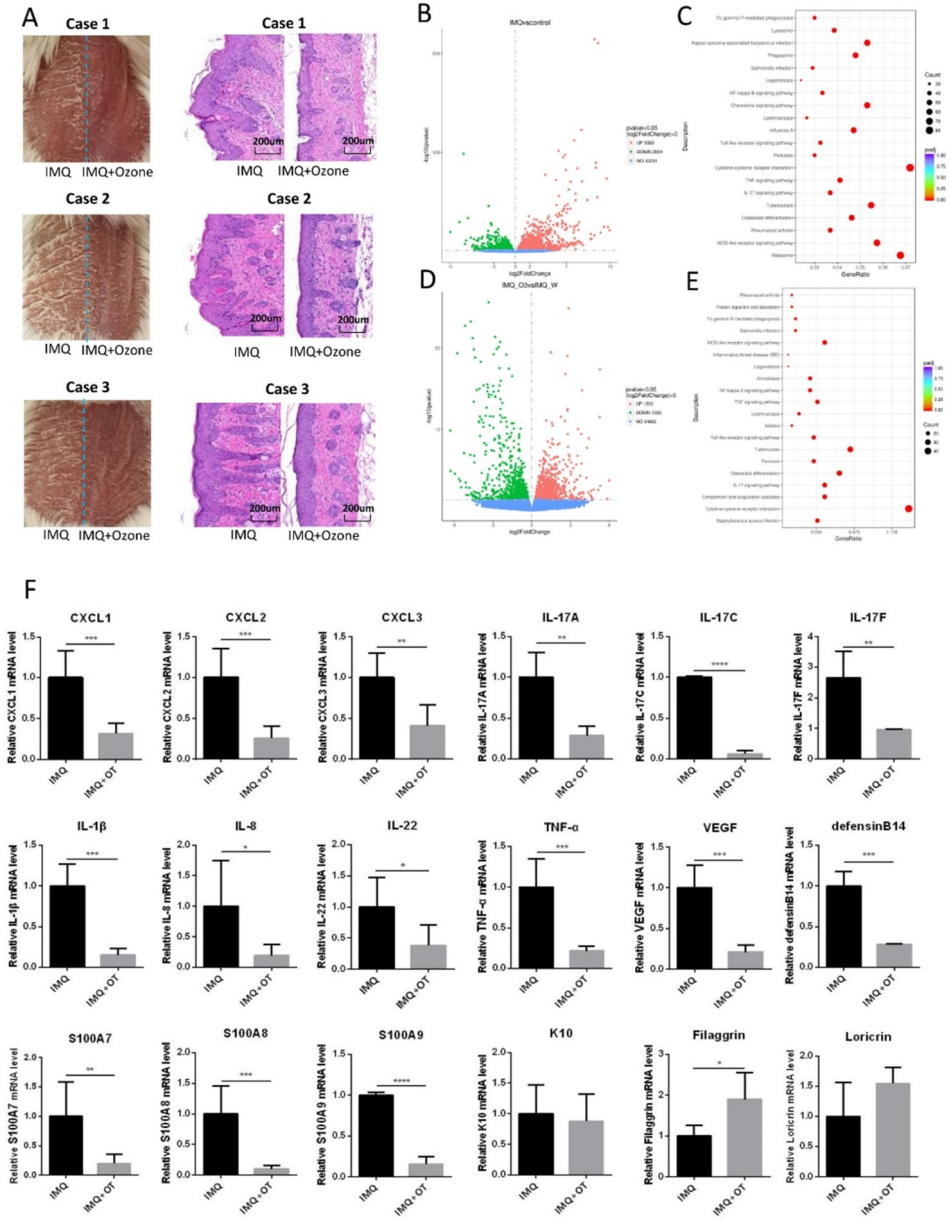
** Topical ozone treatment significantly inhibits IMQ-induced psoriasis- like inflammation. (a)** Psoriasis-like lesions were induced on dorsal skins of mice using IMQ. Topical ozone cream was applied on the right side of the lesion but not on the left side of the lesion. **(b)** Transcriptome analysis showing that IMQ induced an upregulation and downregulation of expression levels of 3063 and 2854 genes, respectively, in lesions as compared with normal skin. **(c)** Enrichment of the upregulated KEGG signaling pathway in the IMQ group versus control group. **(d)** Transcriptome analysis showing that ozone treatment caused an upregulation and downregulation of expression levels of 1203 and 1000 genes, respectively, in lesions as compared with IMQ induction group in the self-control experiment. **(e)** Enrichment of downregulated KEGG signaling pathway in the IMQ+Ozone group versus IMQ group. **(f)** Validation of changes in various psoriasis-associated inflammatory factors using qPCR. Note: * = *P* < 0.05; ** = *P* < 0.01; *** = *P* < 0.001; NS = no statistical significance.

In order to further investigate the mechanism underlying topical ozone treatment for IMQ-induced psoriasis-like lesions, we treated the right side of each lesion with topical ozone and kept the left side of the lesion untreated as a contralateral control (Figure 3a). We analyzed transcriptomes in the skin lesions after ozone treatment by RNA sequencing (RNA-seq) and compared with the skin lesions without treatment. We found that IMQ caused an increase in expression of 3083 genes and decrease in expression of 2854 genes, respectively, as compared with the control (Figure 3b). KEGG pathway enrichment analysis revealed that the inflammatory-related signaling pathways such as NF-κB, TLR, TNF, and IL-17 were significantly activated in the IMQ-induced psoriasis mouse model (Figure 3c). In contrast, topical ozone treatment (right side of the lesion) increased expression levels of 1023 genes and decreased expression levels of 1000 genes, respectively, as compared to the lesions without treatment (the left side of the lesions) (Figure 3d). Interestingly, IMQ-induced activation of NF-κB, TLR, TNF, and IL-17 signaling pathways was significantly inhibited by topical ozone application (Figure 3e). Further quantitative RT-PCR confirmed that topical ozone therapy could significantly inhibit the expression of many chemokines, such as C-X-C motif ligand (CXCL) 1, CXCL2, and CXCL3, and psoriasis-associated inflammatory factors, including IL-17a, IL-17c, IL-17f, IL-1β, IL-8, IL-22, TNF-α, vascular endothelial growth factor (VEGF), defensin B14, S100A7, S100A8, and S100A9 (Figure 3f). These results demonstrate that topical ozone therapy can treat psoriasis lesions via inhibition of the local inflammatory processes.

### Inhibition of TLR2/NF-κB signaling by topical ozone treatment

**Figure 4:**
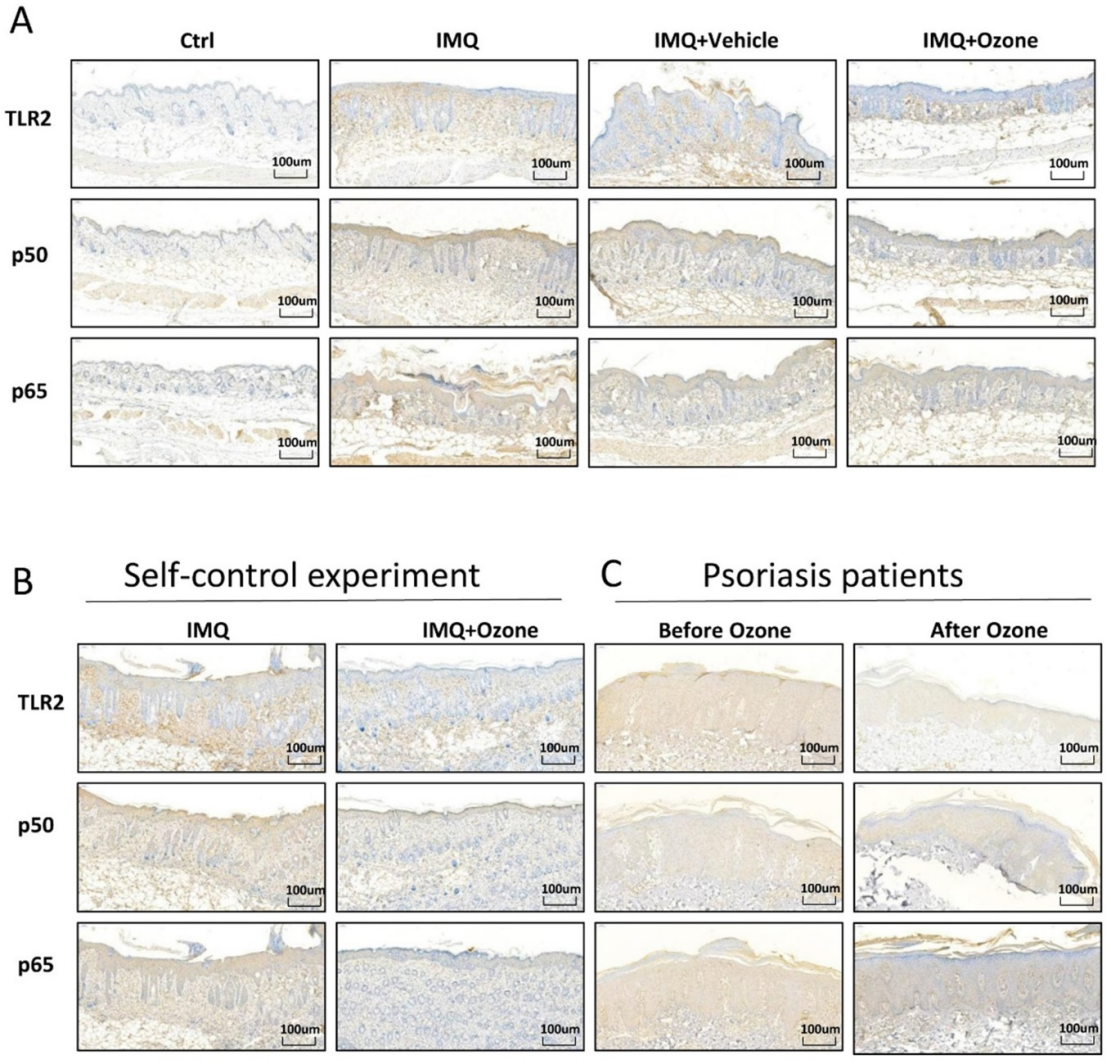
** Topical ozone treatment significantly inhibits TLR2/NF-κB signaling. (a)** Expression levels of TLR2, P50, and P65 in skin lesions from Ctrl, IMQ, IMQ+Vehicle, and IMQ+Ozone groups were evaluated by immunohistochemical analysis. **(b)** Expression levels of TLR2, P50, and P65 in IMQ-induced self-control mouse skin lesions and **(c)** human psoriasis lesions before and after ozone treatment.

Many studies have shown that the TLR2/NF-κB signaling pathway promotes the release of multiple inflammatory factors in psoriasis lesions, aggravating the inflammatory response of psoriasis lesions. In order to examine whether topical ozone could inhibit TLR2/NF-κB signaling, we used immunohistochemistry to characterize the expression profiles of TLR2, P50, and P65 in IMQ-induced psoriasis-like lesions and in patients. We found that topical ozone treatment significantly decreased the IMQ-induced expression levels of TLR2, P50, and P65 in the mouse model (Figure 4a and b) and in patients with psoriasis (Figure 4c). Therefore, topical ozone treatment can significantly inhibit TLR2/NF-κB signaling in psoriatic skin lesions.

### Suppression of Th17 differentiation by topical ozone treatment

**Figure 5:**
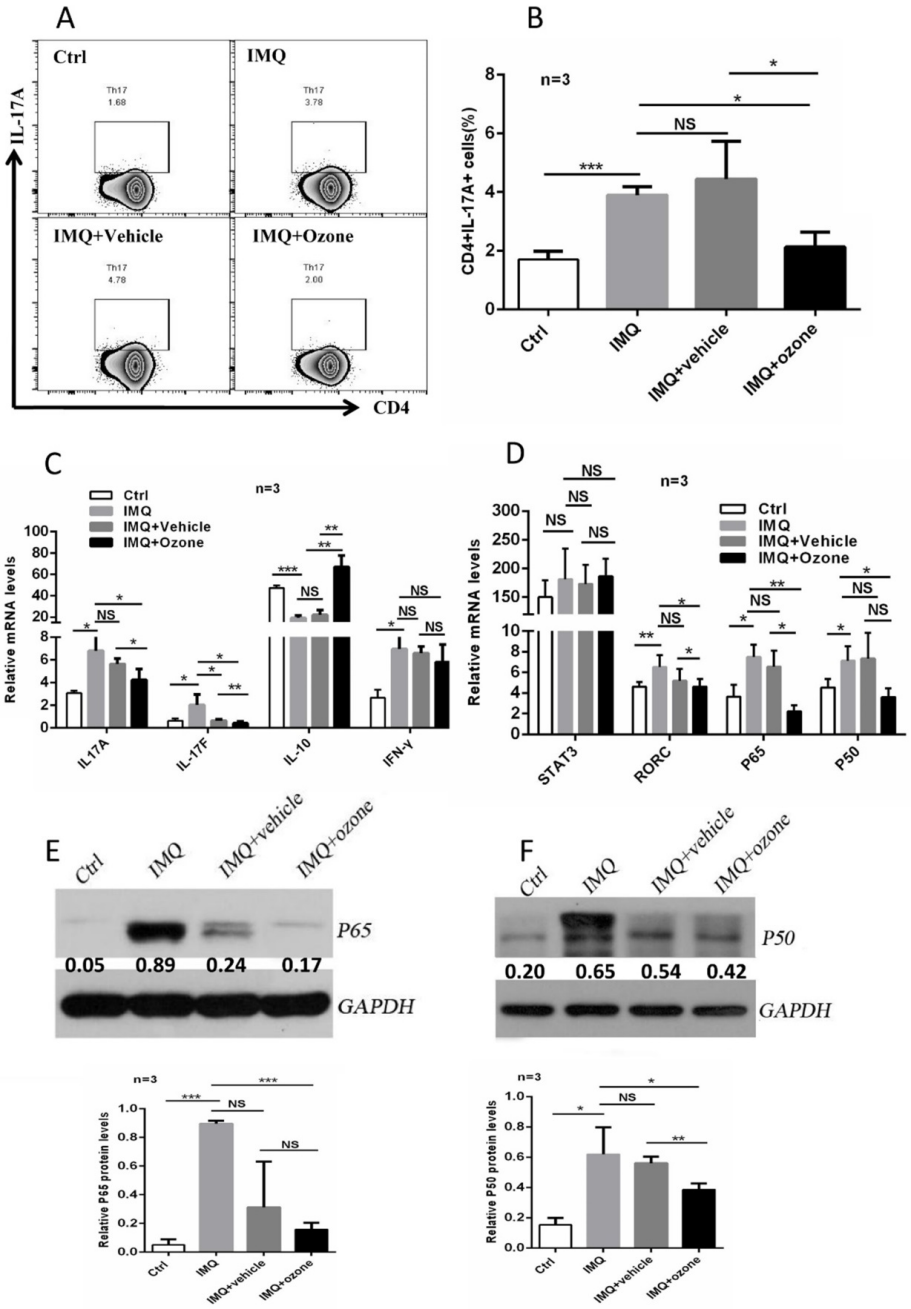
** Topical ozone treatment suppresses T**h**17 differentiation to mitigate psoriasis disease. (a)** Flow cytometry to determine the proportion of Th17 cells in mouse spleens and lymph nodes from four groups, e.g. Ctrl, IMQ, IMQ+Vehicle, and IMQ+Ozone. **(b)** Statistical analysis of the proportion of Th17 cells among different groups. Quantitative PCR to examine expression levels of cytokines **(c)** and transcriptional factors **(d)** in CD4^+^ T cells isolated from mouse spleens and lymph nodes. Western blotting to evaluate expression levels of P65 **(d)** and P50 **(e)** in CD4^+^ T cells isolated from mouse spleens and lymph nodes from four groups. The relative expression levels were normalized to GAPDH levels. Note: * = *P* < 0.05; ** = *P* < 0.01; *** = *P* < 0.001; NS = no statistical significance.

Immune imbalance of CD4^+^ T subset is considered to be a critical factor in the pathogenesis of psoriasis. Evidence has shown that the PASI scores of psoriasis patients are positively related to IL-17 levels in serum and that Th17 cells are the main infiltrating T subset in psoriatic skin lesions 29. Not surprising, ozone therapy resulted in a significant suppression of the IMQ-induced polarized Th17-cell proportions (Figure 5a and b). Ozone treatment significantly inhibited expression levels of T helper lymphocyte-associated cytokines (Figure 5c), including IL-17a, IL-17f, IL-10, and IFN-γ, and key transcription factors (Figure 5d), such as signal transducer and activator of transcription (STAT) 3, RORc, and NF-κB. Ultimately, we examined the expression levels of P50 and P65 in splenic CD4^+^ T cells among the four groups using western blotting. We found that ozone treatment significantly inhibited the expression of NF-κB (Figure 5e and f). Therefore, topical ozone therapy may suppress the IMQ-induced activation of RORc and NF-κB signaling pathways to regulate the differentiation of Th17 cells. Moreover, we also assessed the differentiated proportions of Th1 cells, Th2 cells, and regulatory T (Treg) cells. There was no significant effect on the levels of Th1 and Th2 cells; the proportion of Treg cells was upregulated slightly (Supplementary Figure 1a-c). These data demonstrate that ozone may act on psoriasis mainly by inhibiting the activation and proliferation of Th17 cells and expression of their associated cytokines.

## Discussion

High concentrations of ozone can induce cell damage, leading to respiratory diseases, headaches, skin irritation, and so forth 30-32. However, low concentrations of ozone in the therapeutic range (10-80 μg/ml of gas) can be effectively quenched by the body's powerful antioxidative capacity in order to avoid toxic effects on cells; such concentrations of ozone have been used as a sterilizing agent, promote wound healing, regulate immunity, and carried out analgesic functions 33. Ozone can quickly produce oxygen, reactive oxygen species (ROS), lipid oxidation products (LOPs), and aldehydes when in contact with skin tissue. ROS function as short-acting messengers and quickly disappear; LOPs can enter the blood circulation through lymphatic vessels and capillaries, and function as long-acting messengers. Aldehydes combine with cysteine and glutathione (GSH) to form a stable olefin adduct that can enter various cells of the human body, activating the nuclear factor erythroid 2-related factor 2 (Nrf2)- antioxidant response element (ARE) signaling pathway to improve antioxidant capacity 33. Ozone can play a therapeutic role in many inflammatory diseases due to its antioxidant capacity. For instance, ozone therapy ameliorates inflammation and endometrial injury in rats with pelvic inflammatory diseases 34. Local ozone therapy inhibits the expression of inflammatory factors such as pentraxin-3 (PTX-3), IL-1β, and high-sensitivity C-reactive protein (Hs-CRP) in periodontitis patients 35. We have shown that local ozone therapy not only can kill *Staphylococcus aureus* in atopic dermatitis but also can inhibit inflammation by inhibiting the expression of IL-4 36, 37. An increasing body of evidence suggests that ozone therapy may be able to replace or reduce clinical usages of antibiotics and glucocorticoids, thereby decreasing risks such as antibiotic resistance and side effects from the long-term use of glucocorticoids.

Psoriasis is a chronic relapsing inflammatory skin disease. We posit that ozone therapy can control the progression of psoriasis by inhibiting the inflammatory response of skin lesions. Studies have found that ozonated oil not only delivers reactive oxygen but also maintains therapeutically active ozonated derivatives for a long time 38. Our previous studies have found that ozonated oil is safe and effective for the treatment of stable psoriasis vulgaris, with an efficacy equivalent to that of intermediate-acting glucocorticoids 39. In this study, we have demonstrated that patients' psoriatic skin lesions are mitigated significantly and that inflammatory biomarkers such as IL-17a, IL-6, TNF-α, TGF-β, and IFN-γ are downregulated significantly after ozone treatment. We have also shown that ozone therapy can significantly inhibit inflammatory-related pathways, such as NF-κB, TLR, TNF, and IL-17, in a psoriasis animal model. These data provide an insight into the mechanisms underlying the therapeutic effects of ozone therapy on psoriatic lesions.

Multiple studies have found elevated levels of TLRs in psoriatic lesions 40-43. TLRs are a very important class of pattern recognition receptors (PRRs). After recognizing PAMPs, such as lipopolysaccharides, peptidoglycan, viral products, and bacterial nucleus components, TLRs activate downstream signaling pathways to induce innate immune activation by promoting the release of proinflammatory cytokines and initiating specific immune responses 44-46. Abnormally increased PAMPs (such as lipopolysaccharides and peptidoglycan) in psoriatic lesions activate the NF-κB signaling pathway, promoting the expression of proinflammatory cytokines, which induces a strong inflammatory response in local psoriasis 47, 48. In this study, we have shown that ozone treatment can significantly inhibit the TLR2/NF-κB signaling pathway in psoriatic lesions, thereby attenuating the local inflammatory response of psoriasis. There are a few potential explanations for this. First, ozone therapy can reduce the production of PAMPs by inhibiting colonized microorganisms on the surface of lesions, which reduces the activation of the TLR2/NF-κB pathway. Second, activation of the antioxidant system by ozone such as Nrf2-ARE in the body can antagonize the NF-κB-mediated inflammatory responses 49, 50. Third, oxygen produced by ozone therapy can improve the hypoxic environment of psoriatic skin lesions and inhibit hypoxia-induced inflammatory responses.

The activation of Th17 cells is crucial for the inflammatory response of psoriasis vulgaris lesions 51. Our results show that ozone treatment can significantly inhibit IMQ-induced increases in the number and active function of Th17 cells. Activation of the NF-κB signaling pathway can induce the activation of Th17 cells 52. Therefore, ozone-mediated suppression of the activation of Th17 cells is probably due to the inhibition of NF-κB pathways. In addition to Th17 cells, other immune cells, such as Th1, Th2, dendritic cells (DCs), natural killer (NK) cells, and macrophages, are also involved in the inflammatory response of psoriasis 53. However, our results show that ozone treatment has a minimal effect on these cells. Therefore, ozone likely has a specific effect on the regulation of Th17 cells during the treatment of psoriasis.

In addition to ozonated water and oil, ozone autohemotherapy and ozone gas cavity/acupoint injection have also been reported to improve the body's antioxidant capacity and regulate inflammation 54-56. Whether these treatments can be used for psoriasis has yet to be determined. Ozone can also be used in combination with other agents in order to reduce side effects and increase efficacy. For example, combined intradiscal and periganglionic injection of medical ozone and steroids has a cumulative effect, leading to enhanced overall outcomes in the treatment of pain caused by disk herniation 57. Local ozone therapy has a few side effects, such as irritating pain. It rarely produces systemic side effects. There are some limitations in this study. For example, there are no data regarding the long-term efficacy of the treatment or its impact on recurrence rates. The question of whether ozone treatment plays a regulatory role in the proliferation and differentiation of keratinocytes and in the vasodilation of psoriasis has not yet been answered.

## Supplementary Material

Supplementary figures and tables.

## Abbreviations

HE: hematoxylin and eosin
IMQ: imiquimod
NF-κB: nuclear factor-κB
OAHT: ozone autohemotherapy
PAMP: pathogen-associated molecular pattern
PASI: psoriasis area and severity index
pDC: plasmacytoid dendritic cell
RCM: reflectance confocal microscopy
RORc: retinoid-related orphan nuclear receptor c
TLR2: toll-like receptor 2
