# Supplementary material for: Ozone Therapy Attenuates NF-κB-Mediated Local Inflammatory Response and Activation of Th17 Cells in Treatment for Psoriasis
Source: Int J Biol Sci. 2020 Apr 6;16(11):1833–45. doi: 10.7150/ijbs.41940 (PMC7211165; doi:10.7150/ijbs.41940)
Supplement: Supplementary file 1 — Supplementary figures and tables. [file ijbsv16p1833s1.pdf]

SUPPLEMENTARY MATERIAL

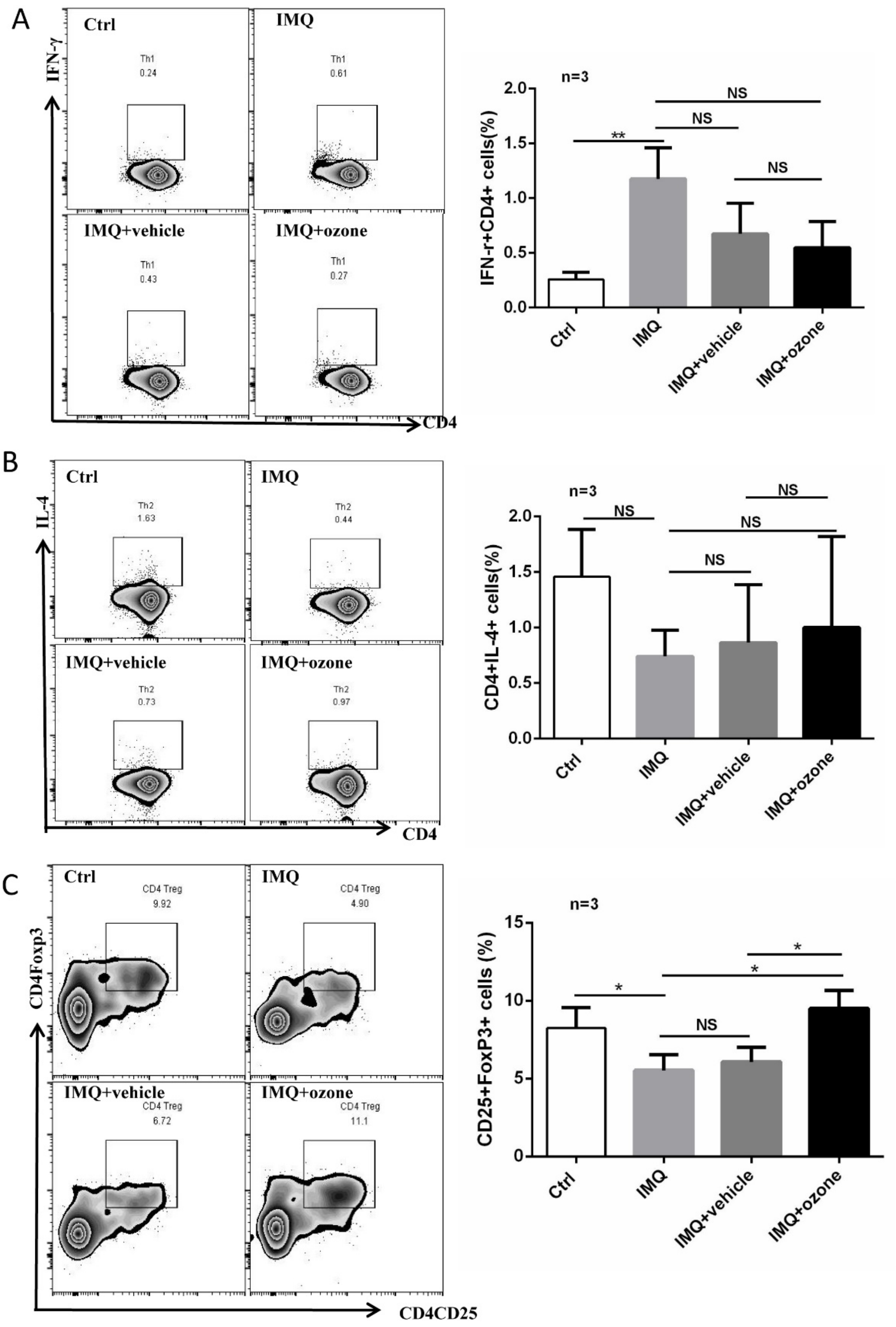

Supplementary Figure 1. Topical ozone therapy does not change the

differentiated proportion of Th1, Th2 and Treg subsets

Proportions of Th1 (a), Th2 (b), and T_{reg} (c) cells from mouse spleens and lymph nodes were assessed by flow cytometry in four groups, e.g. Ctrl, IMQ, IMQ+Vehicle, and IMQ+Ozone.

Note: * = $P < 0.05$; ** = $P < 0.01$; *** = $P < 0.001$; NS = no statistical significance.

Supplementary Table 1. Clinical information of enrollees

No.	Age (years)	Gender	PASI score		
			Before treatment	1 week after treatment	2 weeks after treatment
**1**	35	Male	8.2	6.1	5.0
**2**	42	Male	26.5	14.9	12.6
**3**	26	Male	8	5.6	6.6
**4**	51	Male	25	16.5	12.4
**5**	24	Male	12.4	10.2	8.0
**6**	40	Male	12	7.5	5.0
**7**	34	Female	22.5	17.2	10.5
**8**	49	Male	19	16.5	16.0
**9**	28	Female	18	12.4	9.8
**10**	22	Female	24	15.6	10.9

Supplementary Table 2. Primer sequences for quantitative PCR.

H-IL17A-F:5′-ATTACTACAACCGATCCACCTC-3′	H-IL17A-R:5′-TGGTAGTCCACGTTCCCAT-3′
H-IL17F-F:5′-AGTAAGCCACCAGCGCAACATG-3′	H-IL17F-R:5′-CTCAGAAAGGCAAGCCCCAATA-3′
H-IL6-F:5′-AATTCGGTACATCCTCGACGGC-3′	H-IL6-R:5′-GCCAGTGCCTCTTTGCTGCTTT-3′
H-TNFα-F:5′-GGACACCATGAGCACTGAAAGC-3′	H-TNFα-R:5′-TGCCACGATCAGGAAGGAGAAG-3′
H-TGFβ-F: 5′-GCAACAATTCCTGGCGATAC-3′	H-TGFβ-R: 5′-AAGGCGAAAGCCCTCAAT-3′
H-IFNγ-F:5′-CATCCAAAAGAGTGTGGAGACA-3′	H-IFNγ-R: 5′-TGCTTTGCGTTGGACATTCAAG-3′
H-IL10-F:5′-AAGACCCAGACATCAAGGCG-3′	H-IL10-R:5′-AGGCATTCTTCACCTGCTCC-3′
H-P65-F:5′-AGGGAAATGCCTCTGTGAGCT-3′	H-P65-R:5′-GCAAGAAAGAAGAGGGGTTTGA-3′
H-P50-F:5′-GAGGGAGGCGATCTGATACA-3′	H-P50-R:5′-CTGGACATCAACCTTTCAAGC-3′
H-RORC-F:5′-AGGCCATTCAGTACGTGGTGGA-3′	H-RORC-R:5′-CGTGCGGTTGTCAGCATTGTAG-3′
H-GAPDH-F:5′-ATGGGGAAGGTGAAGGTCG-3′	H-GAPDH-R:5′-GGGGTCATTGATGGCAACAATA-3′
M-Il17a-F:5′-ATGCTGTTGCTGCTGCTGAG-3′	M-Il17a-R:5′-GGAAGTCCTTGGCCTCAGTG-3′
M-Il17f-F:5′-GGAGGTAGCAGCTCGGAAGA-3′	M-Il17f-R:5′-GGAGCGGTTCTGGAATTCAC-3′
M-IL10-F:5'-CTTACTGACTGGCATGAGGA-3'	M-IL10-R:5'-GCATTAAGGAGTCGGTTAGC-3'
M-IFNγ-F: 5'-AGACAATCAGGCCATCAGCA-3'	M-IFNγ-R: 5'-CAACAGCTGGTGGACCACTC-3'
M-RORc-F:5′-GGACAGGGAGCCAAGTTCTCA-3′	M-RORc-R:5′- CACAGGTGATAACCCCGTAGTGG-3′
M-STAT3-F:5'-GTTGGAGCAGCATCTTCAGG-3′	M-STAT3-R:5'-GCATGTCTCCTTGGCTCTTG-3′
M-P50-F: 5′-ATGGTGGTTGGCTTTGCAAA-3′	M-P50-R:5′-TGCACCAGAAGTCCAGGATT-3′
M-P65-F: 5′-TCATCGAACAGCCGAACCAA-3′	M-P65-R: 5′-CAGCCCATAGGGAACTCACC-3′
M-CXCL1-F: 5′-GACTCCAGCCACACTCCAAC-3′	M-CXCL1-R: 5′-TGACAGCGCAGCTCATTG-3′
M-CXCL2-F:5′-AAAATCATCCAAAAGATACTGAACAA-3′	M-CXCL2-R:5′-CTTTGGTTCTTCCGTTGAGG-3′

M-GAPDH-F: 5′-ATGGTGAAGGTCGGTGTGA-3′	M-GAPDH-R: 5′-AATCTCCACTTTGCCACTGC-3′
M-IL-17c-F: 5′-CCTCTAGCTGGAACACAGTGC-3′	M-IL-17c –R:5′-GCGGTTCTCATCTGTGTCG-3′
M-IL-22-F: 5′-TGACGACCAGAACATCCAGA-3′	M-IL-22-R:5′-AATCGCCTTGATCTCTCCAC-3′
M-IL-6-F: 5′-GCTACCAAACTGGATATAATCAGGA-3′	M-IL-6-R:5′-CCAGGTAGCTATGGTACTCCAGAA-3′
M-S100A8-F: 5′-TCCTTGCGATGGTGATAAAA-3′	M-S100A8-R: 5′-GGCCAGAAGCTCTGCTACTC-3′
M-S100A9-F: 5′-GACACCCTGACACCCTGAG-3′	M-S100A9-R: 5′-TGAGGGCTTCATTTCTCTTCTC-3′
M-TNF-α-F: 5′-CTGTAGCCCACGTCGTAGC-3′	M-TNF-α-R: 5′-TTGAGATCCATGCCGTTG-3′
M-VEGF-F: 5′-AAAAACGAAAGCGCAAGAAA-3′	M-VEGF-R: 5′-TTTCTCCGCTCTGAACAAGG-3′
M-CXCL3-F: CCCCAGGCTTCAGATAATCA	M-CXCL3-R: TCTGATTTAGAATGCAGGTCCTT
M-IL-1β-F: GAAATGCCACCTTTTGACAGTG	M-IL-1β-R: TGGATGCTCTCATCAGGACAG
M-IL-8-F: TGCTCAAGGCTGGTCCAT	M-IL-8-R: GACATCGTAGCTCTTGAGTGTCA
M-defensinB14-F: TGAGGCTTCATTATCTGCTATTTG	M-defensinB14-R: TTTCGGAGGGTTTTTGGTAG
M-S100A7-F: GCCTCGCTTCATGGACAC	M-S100A7-R: CGGAACAGCTCTGTGATGTAGT
M-K10-F: GAACAACTTGCAGAAAAGAATCG	M-K10-R: TGTGGTGAGTTCCTTGCTCTT
M-Filaggrin-F: GGACTCTGAGAGGCGATCTG	M-Filaggrin-R: TGCTCCCGAGAAGATCCAT
M-Loricrin-F: GGTTGCAACGGAGACAACA	M-Loricrin-R: CATGAGAAAGTTAAGCCCATCG

Note: H = human; M = mouse; F = forward primer; and R = reverse primer.
